# PlanHab (Planetary Habitat Simulation): the combined and separate effects of 21 days bed rest and hypoxic confinement on human skeletal muscle miRNA expression

**DOI:** 10.14814/phy2.12753

**Published:** 2016-04-25

**Authors:** Eric Rullman, Igor B. Mekjavic, Helene Fischer, Ola Eiken

**Affiliations:** ^1^ Department of Laboratory MedicineClinical PhysiologyKarolinska Institutet and Karolinska University HospitalStockholmSweden; ^2^ Department of CardiologyKarolinska University HospitalStockholmSweden; ^3^ Department of Automation, Biocybernetics and RoboticsJozef Stefan InstituteLjubljanaSlovenia; ^4^ Unit of Environmental PhysiologySwedish Aerospace Physiology CentreKTH Royal Institute of TechnologyStockholmSweden

**Keywords:** Atrophy, transcriptomics, unloading

## Abstract

The study concerns effects of 21 days of sustained bedrest and hypoxia, alone and in combination, on skeletal muscle microRNA (miRNA) expression. It is expected that astronauts undertaking long‐duration missions will be exposed not only to microgravity but also to a hypoxic environment. The molecular machinery underlying microgravity‐induced alterations in skeletal muscle structure and function is still largely unknown. One possible regulatory mechanism is altered expression of miRNAs, a group of noncoding RNAs which down‐regulate many different target genes through increased degradation or translation of their messenger RNA. Thirteen healthy men underwent three 21‐day interventions, interspersed by 4‐month washout periods: horizontal bedrest in normoxia, bedrest in hypoxia, ambulation in hypoxia. The level of hypoxia corresponded to 4000 m altitude. miRNAs from v. lateralis muscle biopsies were analyzed using a microarray covering ≈4000 human miRNAs. Sixteen mature miRNAs were up‐regulated and three down‐regulated after bedrest. The magnitudes of these changes were small and a large portion of the miRNAs affected by bedrest was also differentially expressed after washout periods. In fact, the number of differentially expressed probe sets over time was substantially larger than what could be detected after bedrest. Still, the majority of the miRNAs (let‐7, miR‐15, miR‐25, miR‐199, miR‐133) that were differentially expressed following bedrest, belong to miRNA families previously reported in the context of muscle physiology, in particular to respond to changes in mechanical loading. Since only minor changes in miRNA expression could be detected after bedrest, our data indicate miRNA to play only a minor role in the substantial change in muscle phenotype seen with unloading.

## Introduction

Skeletal muscles exhibit great plasticity and change both function and structural characteristics in response to changes in work demand. Consequently, from the very beginning of the space‐flight era, concern was raised regarding musculoskeletal dysfunction following periods of microgravity‐induced unloading (Asher 1947; Rummel et al. 1975; Adams et al. 2003). The bedrest experimental model was established as an earth‐based analog for studies on effects of microgravity; individuals are requested to maintain a horizontal, or a 6° head‐down tilt position for extended periods. Bedrest studies have examined the musculoskeletal and cardiovascular deconditioning resulting from unloading, and it has been established that the effects of bedrest are similar to those reported after space missions of the same duration (Adams et al. 2003). Thus, microgravity and bedrest alike, induce weekly decreases in maximal strength of the postural knee extensor and plantar‐flexor muscles of about 4–5% along with a considerably slower rate of muscle atrophy corresponding to about 2–3%/week (Adams et al. 1994; Tesch et al. 2004). Thus, bedrest studies have significantly improved our understanding of muscle dysfunction induced by unloading and inactivity as well as the causative mechanism behind muscle atrophy and reduction in force‐generating capacity. Even though this phenotype of bedrest‐induced muscle deconditioning has been fairly well described, the molecular machinery responsible for the deconditioning is still largely unknown. One such possible regulatory mechanism is altered expression of microRNAs (miRNA), a group of noncoding RNAs which down‐regulate many different target genes through increased degradation or decreased translation of their messenger RNA (mRNA) (He and Hannon 2004; Shan et al. 2008).

Noncoding RNA has emerged in recent years as being of functional importance (Timmons 2011). miRNAs are approximately 22‐nucleotide posttranscriptional regulators of gene product abundance, and are able to block the translation of protein‐coding genes (He and Hannon 2004; Shan et al. 2008). Both in vitro experiments and studies in animals and humans have revealed a number of miRNAs that are highly expressed in skeletal muscle (Timmons 2011; Sharma et al. 2014), transcriptionally regulated by myogenic differentiation factors (Rosenberg et al. 2006), able to influence processes in muscle remodeling and known to be induced by exercise and changed in disease that influence muscle structure and function (Chen et al. 2006; Eisenberg et al. 2007; Aoi et al. 2010; Sharma et al. 2014). miRNAs have also been reported to change in response to changes in muscle loading: A study in rats showed that several miRNAs, such as miR‐107, miR‐221, miR‐208b, miR‐499, and miR‐23b, are down‐regulated in the soleus muscle after 2 or 7 days of hindlimb suspension (McCarthy et al. 2009). miRNA has also been analyzed in the vastus lateralis muscle of humans exposed to sustained periods of postural muscle unloading. Thus, Ringholm et al. (2011) reported a 10% decrease in miR‐1 and miR‐133a content after 7 days of seated bedrest. In another study, applying a more exploratory approach, approximately 10 miRNAs were reported to be down‐regulated by as much as 15–30% after 10 days of horizontal bedrest, but miR‐1 and miR‐133 were unaffected (Rezen et al. 2014). Experiments in rodent models have also put forward the let‐7 family of miRNAs as “mechanoMirs,” that is, decreased expression following increased mechanical loading (Mohamed et al. 2015).

Overall, studies utilizing repeated measures in the same subjects have largely reported changes in miRNA expression to be in the range of <20% and data on the direction of these changes are often inconsistent. For instance, miRNA‐1 and miRNA‐133 have been reported to increase by 25% after a single bout of exercise (Nielsen et al. 2010), whereas, Drummond et al. (2008) reported a decrease of 25% in the precursor miRNA‐133a but no change in the mature miRNA‐133 after resistance type exercise. Even though subtle alterations in muscle miRNA abundance may be biologically important, it thus appears that miRNA analysis may be associated with a considerable risk of conducting both type I and II errors.

It is expected that in future planetary habitats astronauts will be exposed not only to reduced gravity but also to hypobaric, hypoxic environments. The current investigation constitutes a part of a larger study (the PlanHab study), the general aims of which were to elucidate biological effects of prolonged musculoskeletal unloading combined with hypoxia. Notably, the aim of the PlanHab study was to perform a basic rather than a high‐fidelity experiment, hence somewhat exaggerating the stimulus conditions, both as regards hypoxia and unloading, compared to those anticipated in future planetary habitats (Bodkin et al. 2006). The present substudy concerns the effects of 21 days of sustained recumbency (bedrest) and hypoxia, alone and in combination, on the miRNA content in the quadriceps femoris muscle. More specifically, the aims were to (1) establish which of the currently known miRNAs are detectable in healthy human skeletal muscle, both across subjects and over time; (2) explore the concerted response of skeletal muscle miRNA expression with bedrest; (3) assess whether hypoxia per se, or in combination with bedrest, affects skeletal muscle miRNA expression; (4) validate both previously reported changes in miRNA expression with bedrest and cross‐validate potential novel findings via repeated interventions in the same subjects.

## Methods

### Test subjects

Healthy men were recruited as test subjects. Inclusion and exclusion criteria have been detailed elsewhere (Debevec et al. 2014). In general, they adhered to those outlined for bedrest studies by the European Space Agency. In addition, individuals who had been exposed to altitudes above 2000 m within 2 months prior to the first experimental campaign were not considered for participation. Following the questionnaires and medical and physiological screening tests, 14 healthy male subjects entered the study and completed the first two experimental campaigns; two subjects dropped out of the study between the second and third campaign for personal reasons, and one subject discontinued his participation prematurely in campaign 3, due to an intestinal problem. Upon study enrollment, their mean ± SD age, body mass, height, and body mass index were 27 ± 6 years, 77 ± 12 kg, 179 ± 3 cm, and 23.7 ± 3.0 kg/m^2^.

All experimental procedures and the study protocol conformed with the principles of the Declaration of Helsinki and were approved by the National Committee for Medical Ethics at the Ministry of Health of the Republic of Slovenia (NCT02293772). Each subject provided informed consent before enrolling in the study.

### Study outline

The experiments and interventions were conducted at the Olympic Sport Centre Planica, located at an altitude of 940 m above sea level, in Ratece, Slovenia. Each subject participated in three experimental campaigns in a counterbalanced fashion: normoxic bedrest (NBR; fraction of oxygen in the ambient gas [FO_2_] = 0.209; inspired partial pressure of oxygen [P_I_O_2_] = 133 ± 0.3 mmHg), hypoxic bedrest (HBR), and hypoxic ambulatory confinement (HAMB), both hypoxic conditions with FO_2_ = 0.141 ± 0.004 and P_I_O_2_ = 90 ± 0.4 mmHg, equivalent to an altitude of ≈4000 m). Each intervention (bedrest or ambulatory confinement) lasted 21 days, and the interventions were separated by a 4‐month washout/recovery period. Several experiments (the majority of which will be reported elsewhere) were performed during the 21‐day intervention periods (D1 to D21), and in particular during the 7 days preceding (baseline days termed: D‐7 to D‐1), and 4 days following each intervention.

### Intervention procedures

During the intervention periods, all subjects were confined to a specific floor of the Olympic Centre, with two subjects in each room. They adhered to strict routines as regards daily activities and dietary intake, as described in detail elsewhere (Debevec et al. 2014). Briefly, energy requirements were estimated on an individual basis using the modified Harris–Benedict resting metabolic rate equation (Hasson et al. 2011). The diet provided a daily protein intake of >1 g protein/kg body mass, and a sodium intake of <3 g/day. Average daily energy intake was 2197 ± 193 kcal in the HAMB condition and 2027 ± 188 and 2018 ± 202 kcal in the NBR and HBR conditions, respectively (Debevec et al. 2014).

One part of the floor was maintained hypoxic by means of a vacuum pressure swing‐adsorption system (b‐Cat, Tiel, The Netherlands).

Throughout both bedrest interventions, each subject remained in a horizontal position at all times. He was allowed one pillow for the head and to occasionally lean on an elbow while eating or being transferred to a gurney. Muscular exercise (e.g., static contractions using the foot board) was prohibited. To ensure that all subjects complied with the requirements of the bedrest protocol, they were continuously surveilled by use of video cameras. During the HAMB confinement, each subject was allowed to move freely within the hypoxic area. To mimic a normal level of physical activity, subjects performed 30 min of low‐intensity exercise twice daily.

### Muscle biopsies

Muscle samples were obtained in the morning after a night's fasting (≥10 h) on days D‐6 and D21; the baseline biopsy (D‐6) was taken in a normoxic environment (air), whereas the D21 biopsies were taken in the prevailing ambient gas environment. The subject was transferred to the laboratory in a supine position and a muscle sample was obtained from the superficial mid‐portion of the vastus lateralis of the nondominant leg. The site was anesthetized by injecting 2–4 mL of 2% lidocaine hydrochloride. Following the application of the anesthetic, a 1–2 cm incision was made to the skin, subcutaneous tissue, and muscle fascia, and the muscle sample was removed using a Rongeur biopsy forceps. The sample was weighed, sectioned, and snap frozen in liquid nitrogen. Samples were kept at −80°C until further processing.

### miRNA analysis

Biopsy amounts sufficient for RNA isolation was available in 72 samples obtained from 13 of the 14 subjects.

Total RNA from 15 mg muscle tissue was prepared by the Trizol method (Invitrogen, Waltham, MA) and quantified spectrophotometrically by absorbance at 260 nm and Agilent Tapestration RNA screen Tape (Santa Clara, CA).

In vitro transcription (IVT) was performed using the Bioarray high yield RNA transcript labelling kit (P/N 900182; Affymetrix, Inc., High Wycombe, Buckinghamshire, UK). Unincorporated nucleotides from the IVT reaction were removed using the RNeasy column (QIAGEN Inc, Venlo, Netherlands). Hybridization, washing, staining, and scanning of the arrays were performed according to the manufacturer's instructions (Affymetrix, Inc.) at the Bioinformatics and Expression Analysis service facility at Karolinska Institutet.

Quality control, normalization, and annotation of probe sets were carried out on Affymetrix Expression Console version 1.4.1. Two samples failed quality control based on low signal on several spike‐in probes. Further means to control the quality of the individual arrays included examination using hierarchical clustering and plotting of log expression signal and probe cell intensity. Finally, all samples were inspected using principal components analysis (PCA) on log2 expression signals.

The probe set‐level intensities of the arrays were normalized using the robust multi‐array analysis method (RMA) and “Detection above Background algorithm” (DABG) was conducted on Affymetrix Expression Console version 1.4.1.

On examination of log expression signals, six samples differed significantly from the mean and this was confirmed on inspection of PCA and cluster analysis where these spurious samples clustered together far aside from the mean and close to the samples with defective spike‐in probes. These samples were excluded from further analysis. In all paired analysis (i.e., pre vs. post), the corresponding sample was also removed from analysis. 9 + 9 observations remained for the NBR intervention and 27 observations (9 + 9 + 9) observations remained to analyze the effect of time/repeated interventions.

Analysis of differential expression was carried out using linear models for microarray data (LIMMA) (Ritchie et al. 2015) on the R statistical software platform version 3.2.1. Correction for multiple hypothesis testing was done by Benjamini and Hochberg correction and a false discovery rate of <10% was deemed significant. All data concerning the microarrays has been deposited at the Gene expression Omnibus, along with all relevant subject data and experimental design, accession number: GSE74469.

## Results

**Figure 1:**
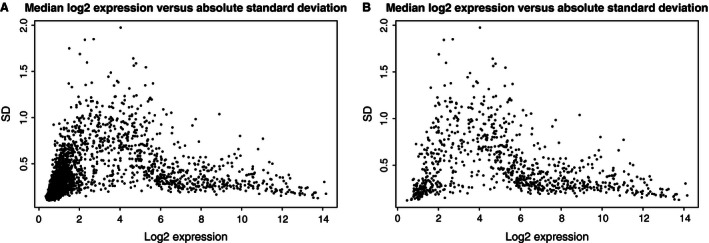
Scatter plot over median log2 expression level versus standard deviation across samples for (A) all probe sets, (B) all probe sets detected above background in >50% of samples. Scatter plots reveal an inverse relationship between expression level and variance, that is, the intersample variability is several fold larger for less abundant probe sets.

The array contained 4603 probe sets annotated as human, this includes both mature and pre‐miRNAs. Of these, 455 probe sets were detected above background in all samples (*P* < 0.05) and 894 probe sets were detected above background in ≥50% of the samples. Another strategy to filter out low abundant or unexpressed probe sets either alone or after DABG‐filtering is by calculating IQR or SD across all samples and discard all probe sets with a low variability across all samples. In this case, IQR‐filtering turned out to discard a large number of well‐established “myomirs” (i.e., miRNA‐133 and miRNA‐1) due to low variability, despite high abundance of the transcripts. In contrast to these highly expressed miRNAs, a large number of miRNAs expressed at a low to modest level (i.e., ≈4–6) displayed a very high absolute variance across samples, whereas most of the highly expressed miRNAs had a stable expression across samples (Fig. 1). Therefore, using intersample variability to filter probe sets was not a fruitful strategy. In fact, the intersample variability can imply a high degree of noise, either biological or technical in nature, among probe sets with an expression in the range of log_2_ ≈2–6. When focusing on miRNAs expressed above the threshold of log_2_ 7 only 253 probe sets remained, which is similar to the numbers detected in previous array studies on skeletal muscle miRNA expression (Gallagher et al. 2010; Drummond et al. 2011). This result also confirms previous reports measuring highly expressed myomirs in vivo – that the interquartile range is less than ±log_2_ 0.5 across samples, which corresponds well to the reported mean changes of about 10–30% with various interventions.

**Figure 2:**
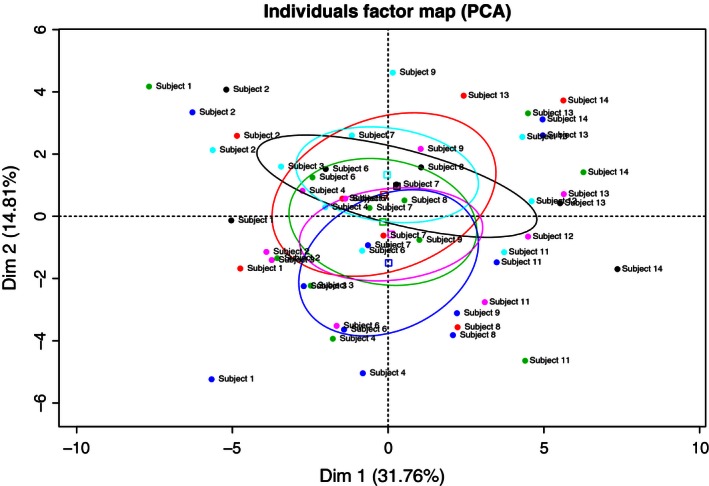
Principal components analysis (PCA) based on probe sets with a median expression level >7 across samples. Most of the observations are located close to origo, illustrating that variance across samples is low. No significant clustering based on either experimental condition or subject is noted. Interventions are color coded, both pre‐ and postbiopsy: Green and blue denote pre‐ and posthypoxic bedrest, respectively. Black and red denote pre‐ and posthypoxic bedrest. Turquoise and purple denote pre‐ and postnormoxic bedrest, respectively. Colored circles illustrate portion of total variance contributed by each condition, the almost complete overlap indicate that variance is shared between the conditions and thus that there was no difference in overall miRNA expression across the six conditions.

In a first attempt to explore, if any of the interventions induced a globally detectable change in miRNA expression, PCA and hierarchical cluster analyses were performed. There was no difference in overall raw probe‐cell intensity or normalized expression levels between the different conditions (*P* = 0.2–0.35, paired *t*‐test). Cluster and PCA analyses of raw log2 expression signals across all probe sets in the QC steps showed a low degree of intraindividual similarity, that is, samples from the same subject was not clustered together. This can be due to the fact that most of the probe sets are unexpressed and therefore a large portion of the total variance in every sample is due to background noise. Thereafter, a PCA analysis was performed using all probe sets annotated as human with a median expression level >7 across all samples: No significant separation based on experimental condition could be detected with PCA (Fig. 2). This is not unexpected, as these analyses take into account the global variance across all measured miRNAs, and only changes of considerable magnitude across a large portion of the measured miRNAs will render a signature distinguishable through cluster or PCA analysis.

**Figure 3:**
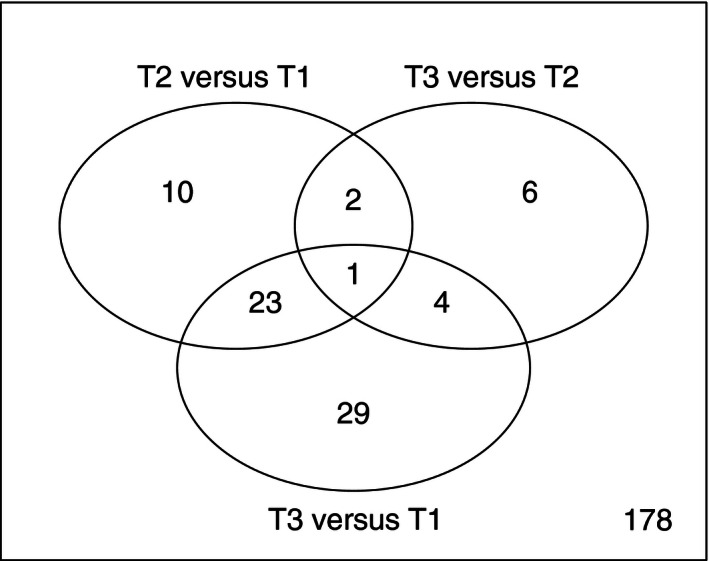
Venn diagram of differentially expressed probe sets over time, that is, when comparing the biopsies taken prior to the three interventions. T1, T2, and T3 denote first, second, and third campaign, respectively, with a 4‐month washout between each intervention. A total of 36 probe sets were differentially expressed at the onset of the second campaign compared with the onset of the first. The majority of these remained differentially expressed when comparing the onset of the third versus the first campaign (T3 vs. T1) and the largest difference is noted in T3 versus T1 (57 probe sets), possible indicating a cumulative effect, that is, the more interventions the larger the difference in the baseline sample.

**Figure 4:**
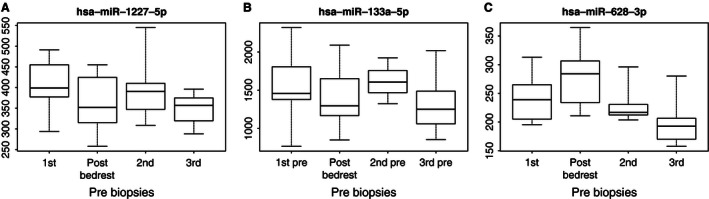
Box plots of the miRNAs differentially expressed after 21 days of bedrest but also differentially expressed over time. Box plots show de‐logged expression values of miRNA expression and display median, quartiles, and range for the first prebiopsy, Post 21 days of bedrest, second and third preintervention biopsy, respectively. (A) MiR‐1227‐5p was significantly down‐regulated following 21 days of bedrest (FDR 4%), and was down‐regulated over time (FDR 8% T3 vs. T1). (B) MiR‐133a‐5p was significantly down‐regulated following 21 days of bedrest (FDR 9%), but was also down‐regulated over time (FDR 8% T3 vs. T1). (C) MiR‐628‐3p was significantly up‐regulated following 21 days of bedrest (FDR 9%), but was significantly down‐regulated over time (FDR 2% T3 vs. T1).

Possible systematic changes between the three baseline samples were addressed through a time‐course model utilizing the order of which the three prebiopsies were used as factor, thus analyzing effects of time and also the accumulated effect of repeated interventions on miRNA expression. This was carried out using LIMMA, which is a flexible tool for analysis of differential expression and allows for very complex factorial design experiments, such as the present experiments. A robust model was constructed in LIMMA adding the order of interventions as factor along with the subject ID to account for the pairwise design: formula ~0 + Time + Subject. Subsequently, the three main contrasts were derived from the model (Time2–Time1, Time3–Time2, and Time3–Time1) and *P*‐values from each contrast were adjusted using Benjamini and Hochberg correction. All probe sets annotated as human (both mature and pre‐miRNA) with a median log2 expression >7 were analyzed. Surprisingly, 78 probe sets were differentially expressed over time, with a cumulative pattern: 35 miRNAs changed their expression between the first and second intervention, and 57 probe sets were differentially expressed at the beginning of the third intervention compared with the first (Figs. 3 and 4). See supplementary file for complete table of differentially expressed probe sets. The study was designed to minimize confounding effects of repeated biopsies and repeated interventions both by balancing the order of the three interventions between subjects, but also through a 4‐month “washout” between interventions. There were two dropouts from the study during the experiments (see methods) and another four had to be excluded due to technical failures in the chip hybridization. Unfortunately, this skewed the balanced study design and in effect the majority of the examined subjects were exposed to NBR prior to the HBR intervention. Therefore, an effect from previous interventions cannot be excluded on the expression levels measured before the HBR intervention.

The main purpose of the study was to explore if any relevant changes in miRNA expression occur with bedrest and since a number of baseline deviations were detectable, we focused the analysis on NBR. To this end, a new robust LIMMA model was constructed analyzing pre‐ versus post bedrest samples with subject added as a factor. All probe sets annotated as human with a median expression <log2 7 were considered, and only arrays which passed all QC steps were used (*n* = 2 × 9). In total, 43 probe sets were found to be differentially expressed after 21 days of bedrest, 16 were up‐regulated, and 27 were down‐regulated at an estimated false discovery rate of <10%. All of the up‐regulated probe sets belonged to mature miRNAs, whereas, the majority (*n* = 15) of the down‐regulated probe sets were annotated as pre‐miRNAs and only three of the down‐regulated probe sets belonged to validated families of miRNA (miR‐133, miR‐1207, and miR‐1227). Intriguingly, three of the miRNAs being differentially expressed after bedrest were also differentially expressed over time (i.e., when comparing the three preintervention samples) and another two were borderline significant (FDR 11%) with regard to changes over time (Table 1 & 2, Figs. 3 and 4).

**Table 1:** miRNAs up‐regulated following 21 days of bedrest

Probe set	miRNA	miR‐family	Fold‐change	AveExpr	FDR adj. *P‐*values	Fold change over time	FDR adj. *P*‐values
20500733	hsa‐miR‐128‐3p	miR‐128	1.4	8.8	0.04	1.2	0.25
20500151	hsa‐miR‐25‐3p	miR‐25	1.4	8.4	0.00	0.9	0.51
20500400	hsa‐miR‐199a‐3p	miR‐199	1.3	9.3	0.05	1.1	0.84
20500458	hsa‐miR‐199b‐3p	miR‐199	1.3	9.3	0.05	1.1	0.84
20500718	hsa‐miR‐15b‐5p	miR‐15	1.3	9.0	0.04	0.9	0.26
20500171	hsa‐miR‐92a‐3p	miR‐25	1.3	12.0	0.04	0.9	0.45
20500137	hsa‐miR‐19b‐3p	miR‐19	1.3	9.0	0.02	1.0	0.99
20504378	hsa‐miR‐628‐3p	miR‐628	1.2	7.9	0.03	0.9	**0.07**
20500715	hsa‐let‐7i‐5p	let‐7	1.2	10.2	0.03	1.0	0.96
20500128	hsa‐miR‐16‐5p	miR‐15	1.2	12.4	0.07	0.9	0.13
20500713	hsa‐let‐7 g‐5p	let‐7	1.2	9.5	0.04	1.0	0.97
20500119	hsa‐let‐7d‐5p	let‐7	1.2	12.6	0.06	0.9	0.11
20500112	hsa‐let‐7a‐5p	let‐7	1.2	12.9	0.01	1.0	0.94
20500730	hsa‐miR‐125b‐5p	miR‐10	1.2	12.3	0.03	1.0	0.62
20500117	hsa‐let‐7c‐5p	let‐7	1.1	12.9	0.03	1.0	0.43
20500115	hsa‐let‐7b‐5p	let‐7	1.1	13.5	0.05	1.0	0.81

Significantly up‐regulated mature, human micoRNAs after 21 days of bedrest with a false discovery rate of <10%. Fold change denotes unlogged fold change after 21 days of bedrest. AveExpr denote geometric mean of the log_2_ expression value across all samples. Fold change over time denotes fold change difference in the prebiopsy at the beginning of the third campaign versus the expression in the prebiopsy taken at the beginning of the first campaign. Bold text signify that the change over time is significant at a false discovery rate of <10%.

**Table 2:** miRNAs down‐regulated following 21 days of bedrest

Probe set	miRNA	miR‐family	Fold change	AveExpr	FDR adj. *P*‐values	Fold change over time	FDR adj. *P*‐value
20506771	hsa‐miR‐1227‐5p	miR‐1227	0.9	8.6	0.04	0.9	**0.08**
20500738	hsa‐miR‐133a‐5p	miR‐133	0.8	10.5	0.09	0.9	0.11
20506801	hsa‐miR‐1207‐5p	miR‐1207	0.8	8.3	0.06	1.0	0.88

Significantly down‐regulated mature, human micoRNAs after 21 days of bedrest with a false discovery rate of <10%. Fold change denotes unlogged fold change after 21 days of bedrest. AveExpr denote geometric mean of the log_2_ expression value across all samples. Fold change over time denotes fold change difference in the prebiopsy at the beginning of the third campaign versus the expression in the prebiopsy taken at the beginning of the first campaign. Bold text signify that the change over time is significant at a false discovery rate of <10%.

## Discussion

The molecular machinery responsible for alterations in skeletal muscle structure and function following exposure to microgravity is still largely unknown, while the phenotype has been fairly well characterized. For instance, there is a weekly loss of muscle mass corresponding to 3% of the lean mass along with a 20% decrease in insulin sensitivity (Mikines et al. 1989; Adams et al. 1994). One possible mechanism behind these profound changes in muscle structure and function following unloading is altered miRNA expression and in this study, we explore if skeletal muscle miRNA expression changes after a 21‐day bedrest intervention using microarrays.

We identified ≈250 miRNAs present in the skeletal muscle of healthy subjects at a similar or higher level of expression as well‐established “myomirs,” in the skeletal muscle of healthy subjects. We further demonstrated that the 21‐day bedrest confinement lead to differential expression in the muscle of 43 probe sets of which, 19 belonged to mature miRNAs. Several of these were differentially expressed also over time, that is, when the subjects returned for the next two interventions. Thus, despite a washout period of 4 months between each intervention, a substantial number of miRNAs were differentially expressed when the subjects returned for the next intervention. In most cases, the direction of the differential expression over time was the opposite of that noted after bedrest. If this is of biological significance, it implies that a change in miRNA expression induced by the bedrest regimen was followed by a reciprocal miRNA change, an effect which presumably was unrelated to the biopsy procedure. Thus, if a change in expression was induced by the first biopsy, one would expect any long‐term trend to be in the same direction. Still, the fact that the amplitudes of the changes caused by 21 days of bedrest were in the order of ≈15%, and that changes of similar magnitude were measured over time, implies a low signal‐to‐noise ratio. A notion that is corroborated by the observation that samples from the same subject did not cluster together on either hierarchical clustering or PCA. This also shows that the within‐subject correlation was low, a pattern that persisted regardless of whether only highly expressed probe sets or all probe sets were analyzed. The low within‐subject correlation was confirmed by calculation of the overall correlation coefficient within subjects over time, which was <0.1. On the other hand, the magnitude of the changes in miRNA expression in this study seems to be in the same range as reported in most other studies measuring skeletal muscle miRNA expression, repeatedly in the same subject (Drummond et al. 2008; Nielsen et al. 2010; Davidsen et al. 2011; Russell et al. 2013). Given the vast number of target mRNAs for each miRNA, even subtle miRNA changes can be expected to have a large impact on phenotype (Timmons 2011). Furthermore, among the 16 up‐regulated miRNAs, the vast majority was represented by several members from the same miRNA‐families. This speaks against a random effect of sampling on a fluctuating baseline, and instead suggests that the findings reflect potentially important changes in miRNA expression with bedrest. A key aim of the study was to investigate possible additive effects of hypoxia. We reasoned that it would be relevant to investigate the interactive effects of hypoxia and unloading not only from a space physiology perspective but also for society in general. Thus, combining chronic hypoxia and bedrest may be considered a model of the basic conditions experienced by the large population of patients suffering from respiratory insufficiency restricting them to a physically inactive lifestyle. Unfortunately, however, it was for several reasons not possible to draw any firm conclusions regarding such interaction. Firstly, due to the number of dropouts and for technical reasons, the counterbalanced study design was skewed, and in 10/13 subjects, the HBR intervention was conducted after the NBR. Secondly, the differences in miRNA expression between the preintervention biopsies made it unmanageable to analyze the HBR samples in a justifiable way, as explained above in the methods section. Accordingly, the analysis focused on the changes in miRNA expression in response to the NBR intervention and over time.

**Figure 5:**
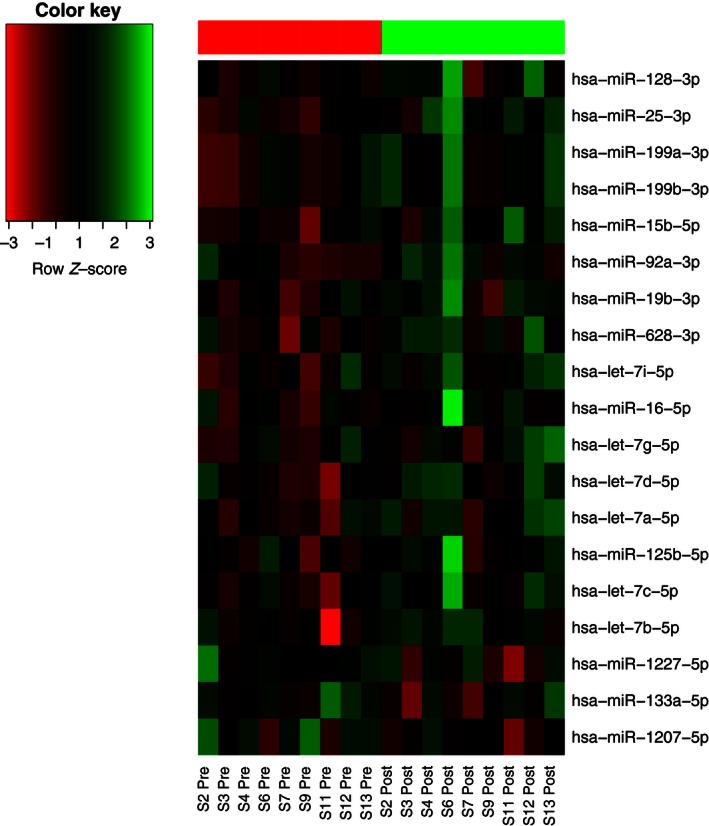
Heat map showing normalized expression levels in individual samples for significantly up‐ and down‐regulated mature, human miRNAs after 21 days of bedrest with a false discovery rate of <10%.

In total, 16 probe sets annotated as mature miRNAs were up‐regulated after 21 days of bedrest (Fig. 5, Table 1). The miR‐family with most strikingly altered pattern of expression was let‐7 where six members were up‐regulated after bedrest. Furthermore, the let‐7 family was among the most abundantly expressed miRNAs detected, in line with previous human array studies and animal models (Gallagher et al. 2010; Zhou et al. 2010; Drummond et al. 2011; Rezen et al. 2014). Up‐regulation of let‐7 miRNA has been reported to lead to impaired insulin signaling and decreased glucose uptake in cultured myoblasts through inhibition of insulin‐receptor expression (Gao et al. 2014) and let‐7 knockdown have also been connected to alleviation of insulin resistance in mice (Frost and Olson 2011; Zhu et al. 2011). Let‐7 –a and –d have also been reported to be increased in patients with type‐2 diabetes (Jiang et al. 2013). In addition, Drummond et al. (2011) reported that several members of the let‐7 family are increased in elderly subjects. A similar finding has been reported in animals (Sun et al. 2015), suggesting that let‐7 may be involved in the aged muscle phenotype including both atrophy and impaired insulin signaling. Consistent with our data, a recent paper by Mohamed et al. (2015) designated the let‐7 family “mechanoMIR” and reported muscle expression of let‐7 family members to be robustly repressed by mechanical loading in rodents. Based on the data on let‐7 being regulated by mechanical loading and influencing insulin resistance and muscle glucose uptake, it is possible that the present up‐regulation of the let‐7 family was caused by the mechanical unloading of the muscle and that it may be involved in the insulin resistance seen after the present (O. EIken and I. Mekjavic, unpubl. data) and previous bedrest interventions (Mikines et al. 1989). In addition to being differentially expressed after bedrest, let‐7d was one of the miRNAs in which the expression fluctuated over time (in the three preintervention biopsies). Albeit speculative, this might suggest that changes in let‐7 expression occurring after one intervention remains for a period longer than the designated 4‐month “washout,” rendering it less responsive to further perturbations. Importantly, the increase noted after NBR was quantitatively subtle, ranging in different subjects from +15 to 25%; differences over time, between the three preintervention biopsies were in the same range. In addition, muscle expression of several let‐7 family members have been reported to decrease slightly after a 10‐day bedrest confinement (Rezen et al. 2014), albeit not significantly.

Accordingly, we conclude that the increased expression of let‐7a,b, and d after 21 days of bedrest was not of greater magnitude than the effects of repeated interventions per se.

Two members of the miR‐15 family (miR‐15b‐5p and miR‐16‐5p) were up‐regulated following bedrest by 25 and 15%, respectively. The human miR‐15 family consists of miR‐15a, miR‐15b, miR‐16, and miR‐195 and the mature miR15a‐5p was actually borderline significantly increased after bedrest (FDR 11%). In a recent twin study, these two miRNAs were shown to be highly differentially expressed when comparing siblings with and without diabetes and also that a major target for miR‐15 family members is the insulin‐receptor and insulin‐receptor substrate, which increased in muscle cell models upon inhibition of miR‐15 (Bork‐Jensen et al. 2015). Thus, we observed a seemingly concerted increase in miR‐15 family expression after bedrest. Based on its experimentally validated effect on insulin‐receptor expression, one might speculate that the miR‐15 family plays a role in the insulin resistance and decreased insulin receptor expression induced by prolonged bedrest (Alibegovic et al. 2010). Another up‐regulated miR‐family with potential implications for diabetes was miR‐199. Both members of the miR‐199 family (miR199‐a‐3p and miR‐199b‐3p) increased by ≈30% after bedrest. These miRNAs have been reported to be down‐regulated in patients with diabetes (Gallagher et al. 2010) and also to be abundantly expressed in the skeletal muscle of pigs where it has been shown to be negatively correlated with muscle mass (Siengdee et al. 2015). To the best of our knowledge, a direct effect of miR‐199 on skeletal muscle protein synthesis or degradation has not been shown to date, but based on the findings in the porcine model, and since muscle atrophy is a hallmark of the bedrest model, the possibility of such connection warrants further investigation.

The two human members of the miR‐25 family (miR‐25‐3p and miR‐92a‐3p), both increased with bedrest. Notably, both these miRNAs have been reported to be down‐regulated in patients with type II diabetes (Bork‐Jensen et al. 2015). The miR‐25 family mainly has target genes related to calcium handling (SERCA, ATP2A2) and angiogenesis (VEGF, CD31, and Tie2) and its inhibition has been shown to increase cardiac contractility (Wahlquist et al. 2014) and circulating levels of miR‐25 have been reported to decrease after 12 weeks of regular endurance training (Nielsen et al. 2014).

Finally, miR‐125b‐5p was significantly elevated after bedrest. miR‐125b is member of the miR‐10 family of miRNAs and in humans it also includes miR‐10, miR‐100, and miR‐99. All of the other members were abundantly expressed but remained stable after bedrest. To date, no study in human subjects has reported changes in skeletal muscle miR‐125 expression, but it has been implicated as an inhibitor of the transcription factor MEF2d in a muscle cell model (Lozano‐Velasco et al. 2015) and miR‐10 and miR‐100 have been reported to be down‐regulated in patients with diabetes (Gallagher et al. 2010).

More probe sets were significantly down than up‐regulated after bedrest but the majority were annotated as pre‐miRNAs, and based on *P*‐values, detection of the down‐regulated probe sets was less certain than detection of the up‐regulated probe sets. Three mature miRNAs were down‐regulated after bedrest: miR‐1207‐5p, miR‐1227‐5p, and miR‐133a‐5p, all of which decreased ≈10–15% (Table 2, Fig. 5). miR‐133 is a well‐established “myoMir” (Chen et al. 2006) mainly known for its involvement in fetal myogenesis and regulation of satellite cell differentiation. Somewhat conflicting data has been put forward regarding skeletal muscle miR‐133 expression and exercise, but down‐regulation has been reported in diabetes (Gallagher et al. 2010), after spaceflight in mice (Allen et al. 2009) as well as after short‐term seated bedrest in humans (Ringholm et al. 2011).

In summary, we investigated the effects of a 21‐day bedrest intervention on skeletal muscle miRNA expression. Only a small number of miRNAs were modestly up‐ or down‐regulated after bedrest. Most of the up‐regulated miRNAs belonged to a number of conserved families, most notably the let‐7 and miR‐15 families. Several of the miRNAs affected by bedrest were also differentially expressed after a 4‐month “washout,” in many cases in the opposite direction. Still, most of the differentially expressed miRNAs have either been reported in the context of muscle physiology (viz. miR‐15, miR‐25, miR‐199) or have been shown to respond to changes in mechanical loading (let‐7) or bed rest‐induced unloading (miR‐133). This indicates that microgravity affects miRNA expression mainly through reduced mechanical load. However, since only minor changes in miRNA expression could be detected after bedrest, our data indicate miRNA to play only a minor role in the substantial change in muscle phenotype seen with unloading.

## Conflict of Interest

The authors have no conflict of interest to declare.
